# A Bibliometric Analysis of Nonspecific Low Back Pain Research

**DOI:** 10.1155/2020/5396734

**Published:** 2020-03-09

**Authors:** Lin-Man Weng, Yi-Li Zheng, Meng-Si Peng, Tian-Tian Chang, Bao Wu, Xue-Qiang Wang

**Affiliations:** ^1^Department of Sport Rehabilitation, Shanghai University of Sport, Shanghai, China; ^2^Department of Rehabilitation Medicine, Shanghai Shangti Orthopaedic Hospital, Shanghai, China

## Abstract

**Background:**

Researchers are highly interested in the study of nonspecific low back pain (NSLBP). However, few have attempted to collect global data, analyze the emerging trends, and conduct reviews from the perspectives of visualization and bibliometrics.

**Purpose:**

We aimed to evaluate research situation and capture subsequent developmental dynamics regarding NSLBP via CiteSpace.

**Methods:**

Publications on NSLBP in recent 19 years were retrieved from the Web of Science Core Collection (WoSCC). We used CiteSpace to analyze publication outputs, document types, countries, institutions, journals, authors, references, and keywords. Knowledge foundation, hot topics, and future direction were then stated.

**Results:**

A total of 1099 papers were collected, and the trend of annual publications maintained growth with small fluctuations. Australia (188) and the University of Sydney (76) were the most prolific country and institution, respectively. The Netherlands (0.84) and the University of Sydney (0.47) had the maximum centrality, thus indicating that they have importance in this field. The journal *Spine* (publication: 87, cocitation counts: 942) ranked first in terms of the volume of publications and cocitation counts. Maher CG (52) who published the most papers and Waddell G (286) who was cited most frequently were the leading authors, thus making strong academic influences. “Motor control exercise” was the largest cluster, which contained most related research articles. 14 references with the strongest citation counts were cited until 2018, thus implying the future development trend. Current hotspots were treatment, meta-analysis, method, and risk factors. Spine, efficacy, adult, and meta-analysis can be regarded as research frontiers.

**Conclusion:**

This study offers insights into the trend of NSLBP to determine major research countries and institutions, core journals, pivotal authors, overall development tendency, hot topics, and research frontiers. Moreover, it will help researchers extract hidden valuable information for further study.

## 1. Introduction

Nonspecific low back pain (NSLBP), without a recognizable pathogeny, has been reported as the major kind of low back pain (LBP) with a high proportion of 90–95% [1, 2]. On a global scale, LBP was the top cause of disability, ahead of other 290 diseases [3–5]. The public health care issues incurred as a result of NSLBP are enormous and costly in most countries [6, 7]. This pain contributes to high pain intensity, depression, functional impairment, and reduced quality of life [8]. Work absence alone caused by NSLBP costs millions of dollars worldwide [9]. As reported, the total costs of LBP are approximately US $100 billion in the USA [10] and AUD $9.17 billion in Australia [11].

In view of the high incidence of NSLBP, a growing number of researchers have studied NSLBP, and relevant articles have been published in academic journals. Nevertheless, few studies on NSLBP have collected global data and conducted a large-scale retrospective analysis through bibliometrics [12–14].

Bibliometrics is a quantitative analysis of published academic literature on a particular topic [15]. Based on citation counts that indicate the impact of a paper on the scientific community, it can conduct an in-depth evaluation of the literature and its references [16, 17]. This analysis is effective and convenient for assessing the productivity of authors, countries, and institutions; identifying geographic distributions and cooperative relations; and uncovering the knowledge structure and development trends [18, 19]. Moreover, the visualization also can excavate valuable information by data-mining technology and display it intuitively [20]. Bibliometric analysis has been widely conducted in various areas, such as neurogenic bladder [21], long noncoding RNA [22], cancer [23], and pain [14, 24–26]. Liang et al. performed a bibliometric analysis to evaluate the emerging trends and hotspots on the area of acupuncture for LBP from 1997 to 2016 [24]. Balague et al. completed systematic analysis and evaluation of NSLBP [6]. On this basis, vital characteristics of this field can be explored via bibliometrics and visualization.

To address the deficiency in quantitative analysis in the study of NSLBP, the aim is to systematically explore the trends of scientific research in this field from 2000 to 2018. Web of Science (WoS) was chosen as the database for mining corresponding literature, and CiteSpace V was applied for visualizing cocitation networks and deep analyzing [27]. Combining citations and inherent relation among literatures, we could obtain some specific influential documents, evaluate current research situation, capture subsequent developmental dynamics regarding NSLBP, and provide researchers with valuable information to facilitate cooperation.

## 2. Methods

### 2.1. Source and Search Strategy

WoS contained abundant information, such as abstracts, references, citation data, and so on, so we chose the Science Citation Index Expanded (SCI-EXPANDED), Social Sciences Citation Index (SSCI), Arts & Humanities Citation Index (A&SCI), and Emerging Sources Citation Index (ESCI) of the Web of Science Core Collect (WoSCC). We used the key words “nonspecific low back pain” and its different expressions as the theme to retrieve relevant literature. Basic information for each article was gathered into text documents, such as countries, institutions, journal sources, authors, and references. The retrieval strategy was as follows: TS=(“non specific low back pain” OR “nonspecific low back pain” OR “non-specific low back pain” OR “nslbp”). No restrictions were imposed on language or document type.

### 2.2. Inclusion Criteria

The literature meets the following criteria: (1) literature published between 2000 and 2018; (2) literature indexed in SCI-E, SSCI, A&SCI, and ESCI of WoSCC; and (3) literature on NSLBP. We performed the data acquisition on December 8, 2018, and collected 1099 papers.

### 2.3. Analysis Tool

CiteSpace V, broadly recognized as a superior scientometric analysis tool, was applied to conduct statistically analyses on the literatures. The analyses drew a series of progressive visualization knowledge domains to detect emerging trends, hidden implications, and landmark literature. On the basis of cocitation maps, we performed cluster analysis and citation burst. In the maps, a large node indicated high occurrence or citation frequency of the object. Different colors represented different years. A node with a red circle in the center, called a burst node, meant that the counts of co-occurrence or citation increased sharply in a certain period. A node with a purple circle in the outline referred to turning points with high centrality and occupied key positions in the knowledge network [27, 28].

## 3. Results and Discussion

### 3.1. Analysis of Publication Outputs

**Figure 1:**
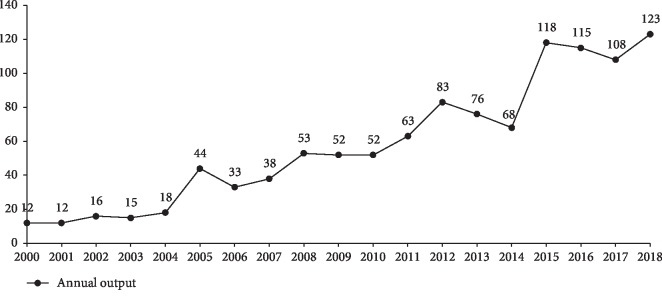
Annual output of NSLBP from 2000 to 2018.

The distribution in the last 19 years of annual publications showed a continuous and unstable rising trend in general. As shown in Figure 1, 2000 to 2004 could be seen as the first phase, and its development trend was steady and barely growing, with the average annual publication being 14.6. 2005 to 2014 could be seen as the second phase, which grew slowly and showed fluctuations, with an average annual publication of 56.2. 2015 to 2018 could be seen as the third phase, which showed a nonobvious changing range, with an average annual publication of 116. The annual output of the third stage was 7.9 times as productive as the first stage. In accordance with the analysis of the line chart, the overall growth trend during the study period predicted a continual increase in the next few years. Additionally, this result indicated that the research intensity is increasing, and researchers developed considerable interest in NSLBP. The publications between 2004 and 2005 and between 2014 and 2015 increased sharply. Some articles published before 2004 and 2014 attracted wide research attention and were cited frequently as the basis, thereby producing significant influence in the field.

### 3.2. Analysis of Document Type

Seven document types existed in total (Table 1). Almost all the papers were written in the form of article, which was the most prevalent document type, accounting for 76.62%. The next was review (13.92%) and editorial material (3.93%). “Non-specific low back pain” was the article that had the most citation frequency. The article mentioned that the all-life morbidity rate of LBP was up to 84% and approximately 1 in 10 people had disability due to LBP [6]. “Diagnosis and treatment of low back pain: A joint clinical practice guideline from the American College of Physicians and the American Pain Society” was the most cited review, which recommended that clinicians needed to advise patients with LBP to keep moving and look after themselves in proactive and appropriate measures [29].

### 3.3. Analysis of Country

Table 2 shows the top 10 countries researching the trends of NSLBP; Australia was the most productive country, accounting for 17.11%, followed by the Netherlands (16.02%) and the USA (14.74%). The top four countries' contributions were all above 10%, which indicated that they contributed major shares in research achievements.

**Figure 2:**
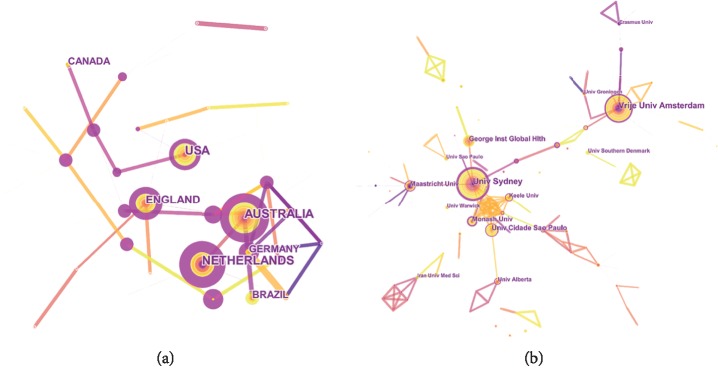
The analysis of countries and institutions. (a) Network map of country cooperation in NSLBP research from 2000 to 2018. (b) Network map of institution cooperation in NSLBP research from 2000 to 2018.

The network map of country cooperation was generated by CiteSpace V with 43 nodes and 50 links (Figure 2(a)). The Netherlands had the maximum centrality (0.84); it was followed by Australia (0.66) and Switzerland (0.66). According to the definition of centrality, these countries showed comparably close collaborations with others and strong academic influence. The comprehensive analysis of publication and centrality indicated that Australia and the Netherlands were in the dominant positions. The Netherlands, Australia, Austria, Bangladesh, Germany, and Switzerland formed Netherland-centered and strong partnerships. Some prolific countries, such as Brazil, Canada, and Belgium, showed no coincidental centrality that the value is 0. Furthermore, they have not performed extensive academic exchanges and cooperation with international counterparts; thus, they occupied a marginal status.

### 3.4. Analysis of Institution

From Table 2, the University of Sydney was the leading institution researching the trends of NSLBP, accounting for 6.92%, followed by Vrije Universiteit Amsterdam (6.46%) and then Maastricht University (3.09%). The top two institutions' proportions were both above 5%, thus indicating that they accomplished relatively substantial research achievements and certain research strengths.

The network map of institution cooperation was generated by CiteSpace V with 216 nodes and 296 links (Figure 2(b)). The University of Sydney showed maximum centrality (0.47). The next was Monash University (0.22) and George Institute for International Health (0.21). These institutions showed a comparably broad range of cooperation with others and a strong academic influence. In terms of publication and centrality, the University of Sydney and Vrije Universiteit Amsterdam were the core strengths and conducted major cooperative position. The strongest partnerships were identified among the University of Sydney, Maastricht University, George Institute for International Health, George Institute for Global Health, and Oslo University Hospital.

### 3.5. Analysis of Journals

Table 3 lists a range of journals that published the most articles on NSLBP from 2000 to 2018. *Spine* accounts for the most outputs (7.92%), followed by *European Spine Journal* (6.19%) and *BMC Musculoskeletal Disorders* (5.82%). In contrast to other journals, the top three journals' proportions were all above 5%, thus signifying that they were authoritative and had a special position. Only one journal's impact factors exceeded 5, whereas the remaining average impact factor was 2.181. Writing in a high-impact-factor journal could be considered challenging. “Exercise therapy for low back pain—A systematic review within the framework of the Cochrane Collaboration back review group” published in *Spine* was cited over 300 times until 2018. This review indicated that exercise therapy might be beneficial to relieve chronic LBP and help patients return to home and society; however, it did not play a notable role in acute LBP by testing the effectiveness of multifarious exercise treatments for participants who had NSLBP with and without radiation into their legs [30]. “Exercise therapy for treatment of non-specific low back pain” in *Cochrane Database of Systematic Reviews*, which was cited the most, also proved the same results that exercise therapy exhibited equal effectiveness with no treatment or conservative treatment for acute LBP, and it exerted slight effects for chronic LBP [31].

**Figure 3:**
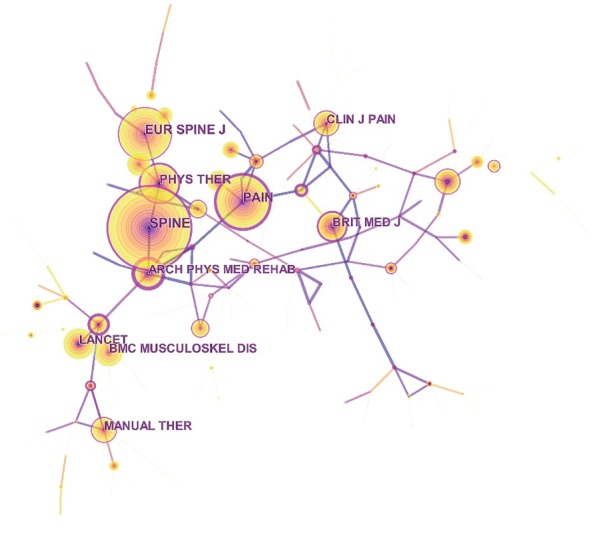
Network map of cocited journals in NSLBP research from 2000 to 2018.

The network map of cocitation journal was generated by CiteSpace V with 187 nodes and 190 links (Figure 3). *Spine* had the maximum cocitation counts (942). The next was *Pain* (644) and *European Spine Journal* (618) (Table 3). The high cocitation counts implied that these journals had superior quality and academic influence relatively and were recognized and undisputed as mainstream. The comprehensive analysis of the publication and cocitation counts showed that *Spine* and *European Spine Journal* were the core journals in the field.

### 3.6. Analysis of Author, Coauthor, and Cocited Author

In Table 4, Maher CG ranks first in terms of publications and accounts for 4.73%, followed by Van Tulder MW (3.46%) and Coast LOP (3.37%). Macher CG and his team explored in accordance with the comparison of the effects of various exercises or physical therapies (such as motor control exercise, spinal manipulative therapy, general exercise, graded activity, and McKenzie method) and placebo in different types of LBP, the influence of depression, the primary care management, and its prognosis.

**Figure 4:**
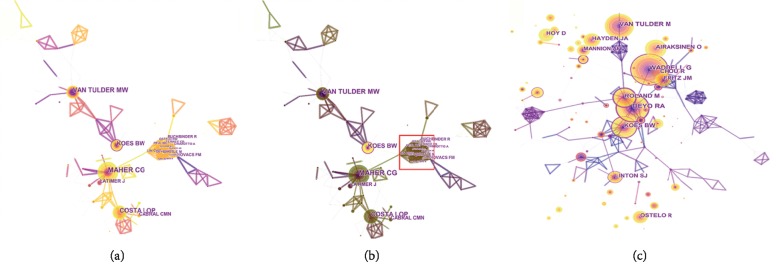
The analysis of authors. (a) Network map of author cooperation in NSLBP research from 2000 to 2018. (b) Network map of author cooperation after spotlight in NSLBP research from 2000 to 2018. (c) Network map of cocited authors in NSLBP research from 2000 to 2018.

The network map of author cooperation was generated by CiteSpace V with 341 nodes and 606 links (Figure 4(a)). On the centrality side, Koes BW had maximum centrality (0.10). The next was Lin CWC (0.09) and Maher CG (0.08). Almost all the authors' centralities were equal to 0, thus reflecting that the collaboration among authors was weak and needed improvement.  Figure 4(b) shows that the lines around nodes in red square were extensive and might mean that these authors generated a small collaboration group. Nevertheless, the area was unhighlighted after spotlight, which demonstrated that our hypothesis that strong exchanges and frequent communications occurred among these authors was wrong.

The network map of cocitation author was generated by CiteSpace V with 250 nodes and 425 links (Figure 4(c)). We also identified the top 10 cited authors according to cocitation counts. Waddell G ranked first (286), followed by Deyo RA (249) and Van Tulder M (226). The high cocitation counts hinted that these authors played a significant role and had a great influence. Their work also accelerated the development of relevant subjects. In terms of the publication and cocitation counts, Koes BW predominated in the research of NSLBP. However, only one prolific author belonged to the top 10 cocited authors, which denoted that their articles did not raise widespread concern. Koes BW and his team studied the efficacy of some interventions (such as exercise therapy, acupuncture, pharmacological treatment, and other complementary and alternative medicine), the management of primary care, risk factors, and prognosis and course in various of aspects. In one highly cited paper, they mentioned that the recommendations of diagnosis and treatment for LBP were roughly similar [32].

### 3.7. Analysis of Cocited References

**Figure 5:**
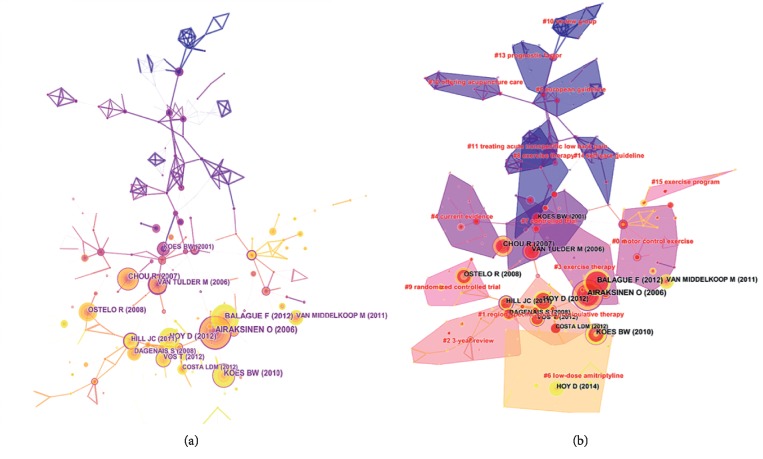
The analysis of references. (a) Network map of cocited references in NSLBP research from 2000 to 2018. (b) Cluster view for cocited references in NSLBP research from 2000 to 2018.

The network map of cocitation reference was generated by CiteSpace V with 279 nodes and 401 links (Figure 5(a)), which clearly delineated scientific relevance in considerable literature. “Chapter 4—European guidelines for the management of chronic nonspecific low back pain” had the maximum cocitation counts (80). Contrary to acute LBP, chronic LBP had fewer guidelines and rather limited therapeutic effect. Encouraging patients with chronic NSLBP by cognitive behavioral interventions is the most promising method [33]. As shown in Table 5, “Non-specific low back pain” was the article which was published recently and cited frequently relatively, mentioning that most of treatments had low effect sizes and patients' preferences or views should also serve as a basis for management [6].

The 10 cocited references listed in the Tables 5 and 6 were fundamental articles, representing knowledge base. According to the cocitation counts and centrality, “Chapter 4—European guidelines for the management of chronic nonspecific low back pain” was the key reference. The article was cited more than 1000 times by the end of 2018, mirroring most people were beset by chronic LBP in contrast to other types. This article studied different diagnoses and treatments to judge which was necessary and beneficial [33]. These top articles revealed that NSLBP arouse researchers' attention, and several people were struggling to search for effective therapeutic methods. As for the management, primary care and exercise training were also followed with interest.

In Figure 5(b), these articles were divided into diverse clusters labeled with title terms extracted from references. The modularity Q score was 0.8613 higher than 0.5, which meant that the definition of every subdomain and the features of knowledge clusters were distinct. The mean silhouette was 0.4993 lower than 0.5 due to the existence of small clusters that reduced the reliability of the value. A high homogeneity to individual clusters remained, which indicated high concentration on various research aspects. “Motor control exercise” was the largest cluster #0; the next was cluster #1 (region-specific spinal manipulative therapy) and cluster #2 (3-year review). Cluster #1 contained most burst references, which implied that “region-specific spinal manipulative therapy” received considerable attention and emphasized research focus in recent years. Two burst cited articles belonged to cluster #3 (exercise therapy), in which its nodes had the largest red circles, and were cited frequently in a long time; hence, exercise therapy was commonly used as remedy over a considerable period of time, probably, because it can improve muscle strength and stability and decrease pain-causing irritants [34].

**Figure 6:**
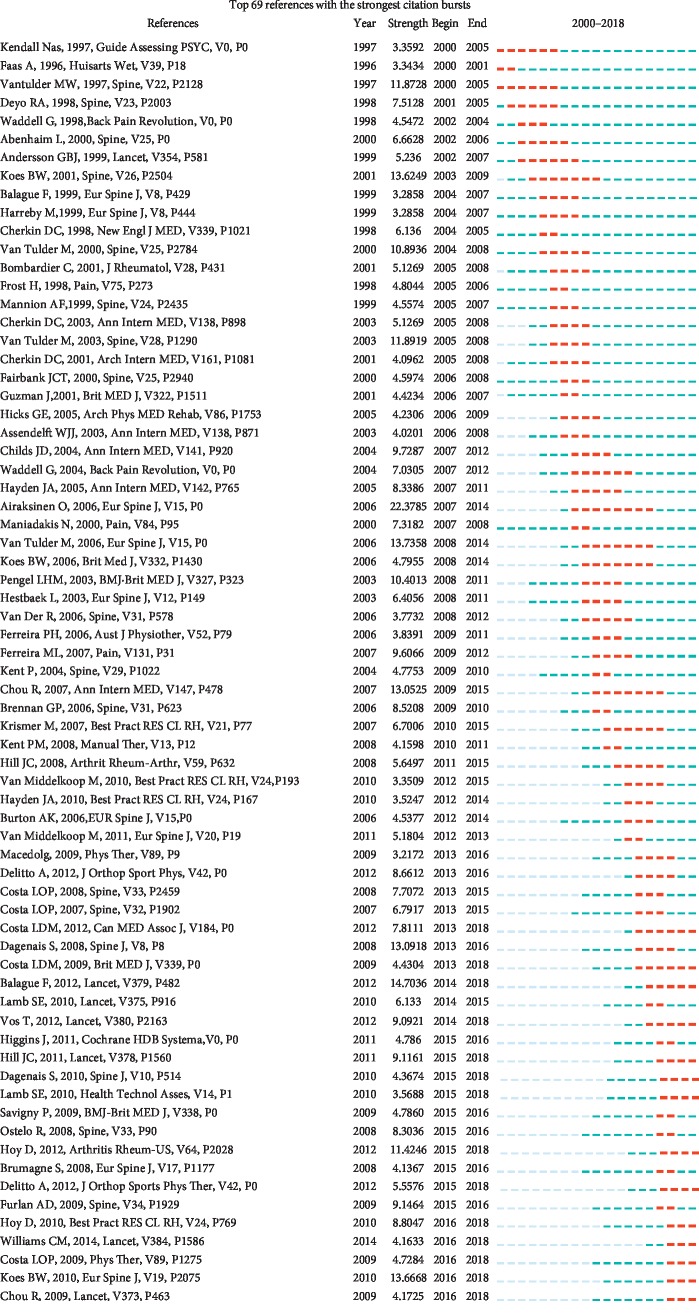
Top 69 references with the strongest citation bursts.

The 14 references were selected in terms of its strongest citation bursts, which continued until 2018 (Figure 6, Table 7), and the citation index also would keep growing rapidly in the upcoming years. In this way, these articles partly indicated current research hotspots. With them as a basis, researchers could go a step further and predict development direction. The topic analysis suggested that meta-analysis, systematic review, and randomized controlled trial were widely applied in research. Scholars showed solicitude for the course of disability and pain in the prognostic process, the efficacy of primary care, motor exercise, cognitive behavioral therapy, and other therapeutic measures for LBP. They also mentioned that physicians should not rely on clinical imaging to diagnose [35].

### 3.8. Analysis of Keyword Co-Occurrence and Burst Keywords

**Figure 7:**
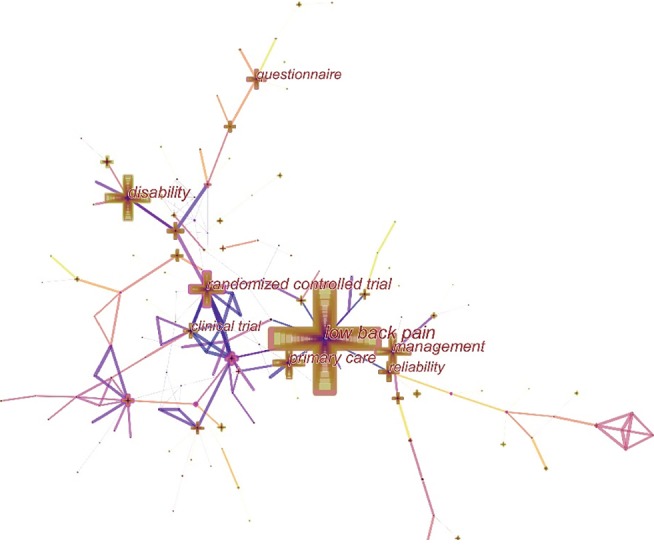
Network map of keyword co-occurrence in NSLBP research from 2000 to 2018.

The network map of keyword co-occurrence was generated by CiteSpace V with 149 nodes and 204 links (Figure 7).  Table 8 presents 40 keywords with the most cocitation counts and centrality. Follow-up, low back pain, randomized controlled trial, risk factor, reliability, management, meta-analysis, exercise, questionnaire, prevalence, and rehabilitation could be regarded as popular hot keywords. Therefore, current hot topics were described.

- Treatment: as a rehabilitation method, exercise therapy is the most effective and broadly concerned. For chronic NSLBP, this therapy can availably relieve pain, decrease disabled degree, and improve the body function. However, for acute NSLBP, it does not have a remarkable effect in relation to no exercise or other conservative treatment [30, 36, 37]. In addition, cognitive behavioral therapy is increasingly popular. The management of primary care also cannot be neglected.
- Meta-analysis: meta-analysis is performed to evaluate the effects of sundry interventions, measure the improvement outcomes of pain and function, and return to work after interfering. This topic is restricted by low-quality literature, heterogeneous data, and inconsistent studies [24, 36, 37].
- Method: randomized controlled trials are often used as research methods to provide evidences on whether treatments have any value. Follow-up, which needs to be taken advantage of, is a significant period to extract data on research findings, in which participants complete questionnaires in many scientific studies [38–40].
- Risk factors: some factors (for instance, age, pain intensity, duration of disease, days of activity limitation, and emotion) may affect the prognosis, lead to severe disability or pain, and develop chronicity [38, 41].

**Figure 8:**
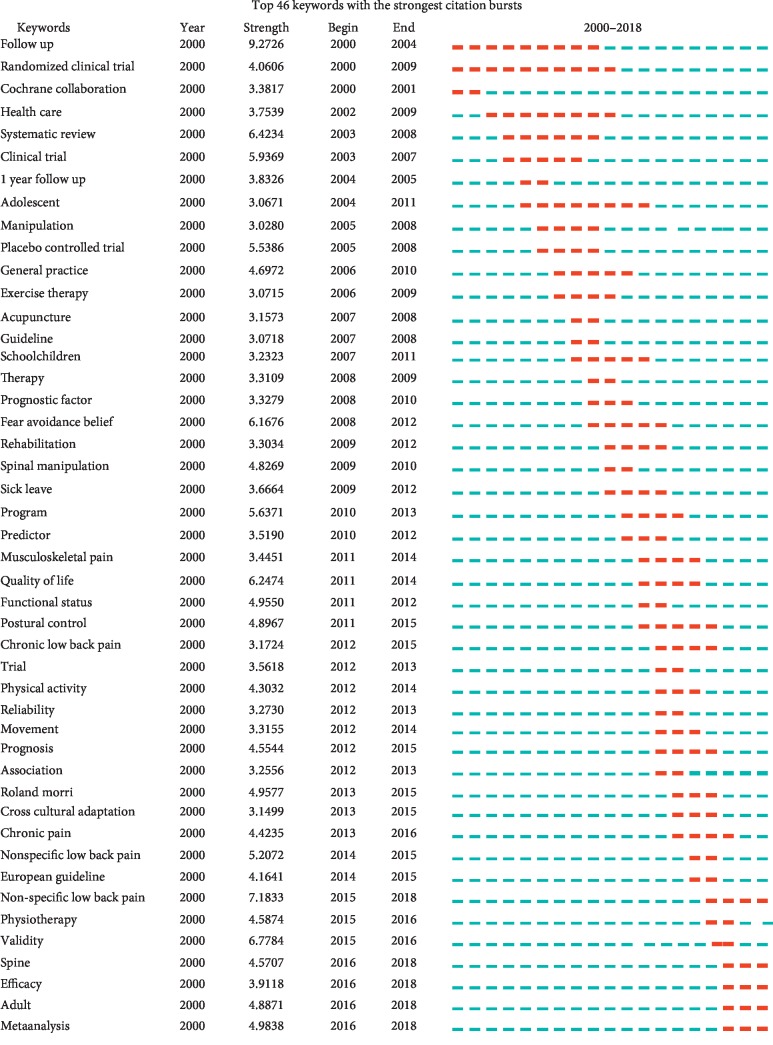
Top 46 keywords with the strongest citation bursts.

On the basis of the distribution of these keywords with the strongest citation bursts, we could predict research frontier. As shown in Figure 8, spine, efficacy, adult, and meta-analysis would be potentially cited frequently over the coming years, which signify the emerging trends. Below were four forefronts in NSLBP:

- Spine: to diagnose the NSLBP or evaluate the efficacy of treatments by various physical and imaging examinations because it is significantly correlated with types of structures in the spine [6].
- Efficacy: to evaluate the effectiveness of various treatments for NSLBP
- Adult: to take adults with NSLBP as research objects in general and the incidence increases with age [41].
- Meta-analysis: to assess the curative effect of treatments versus conventional therapy or no therapy and analyze prognosis

## 4. Conclusion

We can capture valuable information from the bibliometric analysis of the trend of NSLBP between 2000 and 2018. The annual output of related publications continuously grew. Research showed that LBP had a high incidence and a risk of disability. A joint clinical practice guideline recommended that patients needed to perform moderate exercises (moderate-quality evidence). Australia and the Netherlands occupied the dominant positions with main research power, forming cooperative relationship along with Austria, Bangladesh, Germany, and Switzerland. Critical countries and institutions jointly contributed to the development, as well as their strong partnerships. A majority of journals' average IF was <3, which revealed that publishing relevant articles in high-impact-factor journals was challenging, but they still had high cocitation counts. A systematic review found that exercise therapy could obtain a marked effect in treating chronic LBP but not acute LBP. Numerous authors were outstanding in the quantity of research papers rather than quality, lacking communication and cooperation with others. Active authors attempted to evaluate the effectiveness of various exercises or physical therapies and placebos and explore the management of primary care, risk factors, and its prognosis. Randomized controlled trials and meta-analysis were widely applied in research. An article emphasized that cognitive behavioral intervention was the most promising method in chronic NSLBP. As for NSLBP, chronic NSLBP, in particular, bothered many people, and several researchers focused on it. At present, motor control exercise is the most popular method and has drawn the most attention. Relying heavily on clinical imaging to diagnose was not advocated, as mentioned in many studies. Treatment, meta-analysis, method, and risk factors might be the hot topics; spine, efficacy, adult, and meta-analysis might be the research frontiers, indicating renewed trend and future direction.

This study offers an insight into the trend of NSLBP; we can realize major research countries and institutions, core journals, pivotal authors, overall development trend, hot topics, and research frontiers. This study guides further studies to a large extent and may pioneer in this field.

## Figures and Tables

**Table 1:** Types of literature on NSLBP.

Ranking	Document type	Count	Percentage (%)
1	Article	842	76.62
2	Review	153	13.92
3	Editorial material	43	3.93
4	Meeting abstract	31	2.82
5	Letter	22	2.00
6	Proceedings paper	11	1.00
7	Correction	6	0.73

**Table 2:** Top 10 countries and institutions in the study of NSLBP from 2000 to 2018.

Ranking	Country	Publications	Percentage (%)	Centrality	Institution	Publications	Percentage (%)	Centrality
1	Australia	188	17.11	0.66	University of Sydney	76	6.92	0.47
2	Netherlands	176	16.02	0.84	Vrije Universiteit Amsterdam	71	6.46	0.20
3	USA	162	14.74	0.33	Maastricht University	34	3.09	0.14
4	England	141	12.83	0.49	Universidade Cidade de Sao Paulo	32	2.91	0.14
5	Brazil	91	8.28	0.00	George Institute for Global Health	31	2.82	0.09
6	Germany	84	7.64	0.50	Monash University	25	2.28	0.22
7	Canada	81	7.37	0.00	Keele University	20	1.82	0.11
8	Switzerland	66	6.01	0.66	University of Groningen	20	1.82	0.02
9	Spain	65	5.91	0.02	Erasmus University	19	1.73	0.03
10	Belgium	50	4.55	0.00	University of Alberta	18	1.64	0.13

**Table 3:** Top 10 journals and cocited journals in the study of NSLBP from 2000 to 2018.

Ranking	Journal	Publication	Percentage (%)	IF (2018)	Cited journal	Cocitation counts
1	*Spine*	87	7.92	2.792	*Spine*	942
2	*European Spine Journal*	68	6.19	2.634	*Pain*	644
3	*BMC Musculoskeletal Disorders*	64	5.82	1.998	*European Spine Journal*	618
4	*Journal of Back and Musculoskeletal Rehabilitation*	29	2.64	0.982	*Physical Therapy*	472
5	*Manual Therapy*	27	2.46	2.330	*Lancet*	391
6	*Physical Therapy*	27	2.46	2.587	*British Medical Journal*	363
7	*Journal of Manipulative and Physiological Therapeutics*	24	2.18	1.426	*Clinical Journal of Pain*	349
8	*Cochrane Database of Systematic Reviews*	20	1.82	6.754	*Archives of Physical Medicine and Rehabilitation*	341
9	*Archives of Physical Medicine and Rehabilitation*	17	1.55	3.077	*BMC Musculoskeletal Disorders*	341
10	*Journal of Rehabilitation Medicine*	17	1.55	1.802	*Manual Therapy*	316

**Table 4:** Top 10 authors and cocited authors in the study of NSLBP from 2000 to 2018.

Ranking	Author	Publications	Percentage (%)	Centrality	Cited author	Cocitation counts
1	Maher CG	52	4.73	0.08	Waddell G	286
2	Van Tulder MW	38	3.46	0.08	Deyo RA	249
3	Costa LOP	37	3.37	0.04	Van Tulder M	226
4	Koes BW	37	3.37	0.10	Koes BW	194
5	Latimer J	21	1.91	0.00	Roland M	179
6	Kool J	19	1.73	0.00	Airaksinen O	164
7	Kovacs FM	14	1.27	0.03	Chou R	143
8	Underwood M	14	1.27	0.01	Fritz JM	143
9	Bachmann S	13	1.18	0.00	Hayden JA	131
10	Cabral CMN	13	1.18	0.00	Ostelo R	122

**Table 5:** Top five cocited references in the study of NSLBP from 2000 to 2018.

Ranking	Cited reference	Cocitation counts	Publication year
1	Chapter 4—European guidelines for the management of chronic nonspecific low back pain	80	2006
2	Non-specific low back pain	71	2012
3	An updated overview of clinical guidelines for the management of non-specific low back pain in primary care	57	2010
4	Diagnosis and treatment of low back pain: A joint clinical practice guideline from the American college of physicians and the American pain society	55	2007
5	A systematic review of the global prevalence of low back pain	55	2012

**Table 6:** Top five cocited references in terms of centrality in the study of NSLBP from 2000 to 2018.

Ranking	Cited reference	Centrality	Publication year
1	Chapter 3—European guidelines for the management of acute nonspecific low back pain in primary care	0.57	2006
2	Meta-analysis: exercise therapy for nonspecific low back pain	0.46	2005
3	Clinical importance of changes in chronic pain intensity measured on an 11-point numerical pain rating scale	0.44	2001
4	Chapter 4—European guidelines for the management of chronic nonspecific low back pain	0.38	2006
5	Motor control exercise for chronic low back pain: a randomized placebo-controlled trial	0.36	2009

**Table 7:** Fourteen references with the strongest citation bursts that continue until 2018.

Ranking	References	Publication year	Beginning year	Ending year
1	The prognosis of acute and persistent low-back pain: a meta-analysis	2012	2013	2018
2	Prognosis for patients with chronic low back pain: inception cohort study	2009	2013	2018
3	Non-specific low back	2012	2014	2018
4	Years lived with disability (YLDs) for 1160 sequelae of 289 diseases and injuries 1990–2010: a systematic analysis for the Global Burden of Disease Study 2010	2012	2014	2018
5	Comparison of stratified primary care management for low back pain with current best practice (STarT Back): a randomised controlled trial	2011	2015	2018
6	Synthesis of recommendations for the assessment and management of low back pain from recent clinical practice guidelines	2010	2015	2018
7	A multicentred randomised controlled trial of a primary care-based cognitive behavioural programme for low back pain. The Back Skills Training (BeST) trial	2010	2015	2018
8	A systematic review of the global prevalence of low back pain	2012	2015	2018
9	Patellofemoral pain: proximal, distal, and local factors, 2nd International Research Retreat.	2012	2015	2018
10	The epidemiology of low back pain	2010	2016	2018
11	Efficacy of paracetamol for acute low-back pain: a double-blind, randomized controlled trial	2014	2016	2018
12	Motor control exercise for chronic low back pain: a randomized placebo-controlled trial	2009	2016	2018
13	An updated overview of clinical guidelines for the management of non-specific low back pain in primary care	2010	2016	2018
14	Imaging strategies for low-back pain: systematic review and meta-analysis	2009	2016	2018

**Table 8:** Top 20 keywords in terms of frequency and centrality in the study of NSLBP from 2000 to 2018.

Ranking	Keyword	Frequency	Keyword	Centrality
1	Low back pain	482	Follow up	0.99
2	Disability	214	Low back pain	0.95
3	Randomized controlled trial	180	Randomized controlled trial	0.75
4	Management	177	Risk factor	0.58
5	Primary care	171	Prognosis	0.52
6	Questionnaire	107	Reliability	0.36
7	reliability	107	management	0.35
8	Clinical trial	100	Intervention	0.32
9	Exercise	99	Adolescent	0.32
10	Rehabilitation	92	Strength	0.32
11	Therapy	70	Epidemiology	0.28
12	Risk factor	68	Balance	0.27
13	Guideline	68	Metaanalysis	0.25
14	Prevalence	67	Exercise	0.23
15	Follow up	66	Nonsteroidal antiinflammator	0.22
16	Lumbar spine	65	Questionnaire	0.20
17	Physical therapy	55	Prevalence	0.18
18	Metaanalysis	54	Rehabilitation	0.16
19	Back pain	50	Coordination	0.15
20	Classification	46	Placebo controlled trial	0.14

## Data Availability

The data used to support the findings of this study are included within the article.
